# Long-term effect of exposure to lower concentrations of air pollution on mortality among US Medicare participants and vulnerable subgroups: a doubly-robust approach

**DOI:** 10.1016/S2542-5196(21)00204-7

**Published:** 2021-10

**Authors:** Mahdieh Danesh Yazdi, Yan Wang, Qian Di, Weeberb J Requia, Yaguang Wei, Liuhua Shi, Matthew Benjamin Sabath, Francesca Dominici, Brent Coull, John S Evans, Petros Koutrakis, Joel D Schwartz

**Affiliations:** Department of Environmental Health (M Danesh Yazdi PhD, Y Wang ScD, Q Di ScD, W J Requia PhD, Y Wei PhD, L Shi ScD, J S Evans ScD, Prof P Koutrakis PhD, Prof J D Schwartz PhD), Department of Biostatistics (Y Wang, M B Sabath MA, Prof F Dominici PhD, Prof B Coull PhD), and Department of Epidemiology (Q Di, Prof J D Schwartz), Harvard TH Chan School of Public Health, Boston, MA, USA; Vanke School of Public Health, Tsinghua University, Beijing, China (Q Di); School of Public Policy and Government, Fundação Getúlio Vargas, Brasília, Brazil (W J Requia); Gangarosa Department of Environmental Health, Rollins School of Public Health, Emory University, Atlanta, GA, USA (L Shi)

## Abstract

**Background:**

Long-term exposure to air pollution has been linked with an increase in risk of mortality. Whether existing US Environmental Protection Agency standards are sufficient to protect health is unclear. Our study aimed to examine the relationship between exposure to lower concentrations of air pollution and the risk of mortality.

**Methods:**

Our nationwide cohort study investigated the effect of annual average exposure to air pollutants on all-cause mortality among Medicare enrolees from the beginning of 2000 to the end of 2016. Patients entered the cohort in the month of January following enrolment and were followed up until the end of the study period in 2016 or death. We restricted our analyses to participants who had only been exposed to lower concentrations of pollutants over the study period, specifically particulate matter less than 2·5 μg/m³ in diameter (PM_2·5_) at a concentration of up to 12 μg/m³, nitrogen dioxide (NO_2_) at a concentration of up to 53 parts per billion (ppb), and summer ozone (O_3_) at concentrations of up to 50 ppb. We adjusted for two types of covariates, which were individual level and postal code-level variables. We used a doubly-robust additive model to estimate the change in risk. We further looked at effect-measure modification by stratification on the basis of demographic and socioeconomic characteristics.

**Findings:**

We found an increased risk of mortality with all three pollutants. Each 1 μg/m³ increase in annual PM_2·5_ concentrations increased the absolute annual risk of death by 0·073% (95% CI 0·071–0·076). Each 1 ppb increase in annual NO_2_ concentrations increased the annual risk of death by 0·003% (0·003–0·004), and each 1 ppb increase in summer O_3_ concentrations increased the annual risk of death by 0·081% (0·080–0·083). This increase translated to approximately 11 540 attributable deaths (95% CI 11 087–11 992) for PM_2·5_, 1176 attributable deaths (998–1353) for NO_2_, and 15 115 attributable deaths (14 896–15 333) for O_3_ per year for each unit increase in pollution concentrations. The effects were higher in certain subgroups, including individuals living in areas of low socioeconomic status. Long-term exposure to permissible concentrations of air pollutants increases the risk of mortality.

**Funding:**

The US Environmental Protection Agency, National Institute of Environmental Health Services, and Health Effects Institute.

## Introduction

For the past 30 years, many studies have looked at the health effects of air pollution. Long-term exposure to air pollution has been linked to an increase in the risk of death.^1–6^ These studies have led to the creation of national air-quality standards by the US Environmental Protection Agency (EPA). The US EPA regulates six criteria air-pollutants, including carbon monoxide (CO), lead (Pb), sulfur dioxide (SO_2_), particulate matter less than 10 μg per m³ in diameter (PM_10_), particulate matter less than 2·5 μg per m³ in diameter (PM_2·5_), nitrogen dioxide (NO_2_), and ozone (O_3_). The focus of our study was PM_2·5_, NO_2_, and O_3_. Air-pollution standards limit the annual average concentration of ambient PM_2·5_ to 12 μg per m^3^ and NO_2_ to 53 parts per billion (ppb). O_3_ does not have a standard for long-term exposure.^7^ However, whether the standards are sufficient to protect human health, as required by the Clean Air Act remains unclear.

Studies^4,8–12^ have suggested that the adverse effects caused by air pollution follow a no-threshold dose-response curve, and that people exposed to concentrations that are lower than those recommended by guidelines might still have an increased risk of morbidity and mortality. However, most of these studies did not focus on individuals who were always exposed to lower concentrations during study follow-up. Further, by including exposure to higher concentrations, these studies might have been susceptible to exposure-measurement error at high concentrations, for which measured values are rare. In addition, some researchers have argued that measurement error at higher concentrations can bias the shape of the exposure-response curve at lower concentrations, and by excluding all people who were ever exposed to 12 μg per m^3^ PM_2·5_ or higher, we can avoid this problem. Moreover, these studies generally derived effect estimates on the multiplicative scale,^4,8–12^ which are typically non-collapsible and conditional on the distribution of covariates, and rarely used causal modeling methods.^13–15^

To address these gaps, we aimed to examine the relationship between long-term exposure to PM_2·5_, NO_2_, and O_3_, and mortality among all Medicare enrollees who were only exposed to lower concentrations for the full duration of follow-up, using a doubly-robust causal modelling technique that provides unbiased estimates if either method of covariate control is correctly specified.

## Methods

### Study population

We designed this study as a nationwide cohort study. Our cohort included all patients aged at least 65 years enrolled in Medicare in the USA from beginning 2000 to end 2016. Medicare is a national insurance programme for US residents that primarily covers the insurance of older people (≥65 years of age). Medicare beneficiaries made up between 13% and 16% of the total population between 2000 and 2016 in the USA^16^ and 97% of older people in the year 2000.^17^ Patients entered the cohort in the month of January of the year following enrolment and were followed up until the end of study period in 2016 or death. Our study was approved by the IRB at Harvard TH Chan School of Public Health.

### Exposure assessment

We established the level of exposure to PM_2·5_, NO_2_, and O_3_ on the basis of estimates generated from spatiotemporal ensemble models.^18–20^ Briefly, predictors were extracted from satellite-based measurements, land-use data, meteorological data, and chemical-transport models. The predictors were used as input for three machine-learning algorithms, including a random forest, a gradient-boosting machine, and a neural network. We put the predictions generated by these algorithms into a geographically weighted generalised additive model, which in turn produced daily predictions for the contiguous USA, between 2000 and 2016 on a 1 km^2^ spatial scale. We averaged predictions across days in each calendar year to obtain annual averages, and aggregated grid cells to postal codes and assigned exposures to individuals on the basis of their residential postal code in each year. All models showed strong performance, with ten-times cross-validation *R*^2^ values of 0·89 for PM_2·5_, 0·84 for NO_2_, and 0·86 for O_3_.^18–20^ We specifically used warm-season O_3_ concentrations, from April 1, to Sept 30, given that this season is associated with higher average concentrations. All references to O_3_ in this Article refer to warm-season O_3_.

We limited our dataset to individuals who were exposed to concentration that were lower than the maximum recommended by US regulations for all years of follow-up. For PM_2·5,_ that threshold is 12 μg per m^3^, although the calculation of the EPA involves taking the average of annual concentrations over 3 years at each monitoring station. The maximum recommended concentration for NO_2_ is 53 ppb. O_3_ does not have regulations for long-term exposure, so we chose 50 ppb to have enough observations.

We further dropped the lowest three percentile for each exposure. Such low exposures are rare, and modelling is not as accurate when there are scarce measured values to train the models.

### Outcome assessment

We obtained information on all-cause mortality from the Medicare denominator file.

### Covariate assessment

We adjusted for two types of covariates in both the propensity-score model and the outcome regression, which were individual level and postal code-level (ie, ZIP code-level) variables. Individual level variables included age, sex, race, and Medicaid eligibility (supplemental insurance for individuals with low incomes). These data were obtained from the Medicare-denominator file and were updated annually.

We derived postal code-level variables from data generated by the US Decennial Census and the American Community Survey in 2000, 2010, 2011, 2012, 2013, 2014, 2015, and 2016. Values for the remaining years were obtained through interpolation. The variables we included in our models were population density, percentage of the population older than 65 years living below the poverty line, percentage of the housing occupied by the owners, median value of the housing occupied by the owners, median household income, percentage of the population listed as Black, percent of the population listed as Hispanic, and percentage of the elderly population who did not graduate from high school. We further derived information on the percentage of the population who have ever smoked and the mean body-mass index of the respondents at the county level from the Behavioral Risk Factor Surveillance System. We used the Dartmouth Health Atlas to obtain information on the proportion of Medicare beneficiaries with at least one haemoglobin-A1c test in a year, the proportion of beneficiaries with diabetes older than 65 years who had a lipid-panel test in a year, the proportion of beneficiaries who had an eye examination in a year, the proportion beneficiaries with at least one ambulatory doctor’s visit in a year, and the proportion of female beneficiaries who had a mammogram over a 2 year period. We obtained information on the rate of lung cancer in the population from hospital admission data in the Medicare Provider Analysis and Review (MEDPAR) dataset. We also included information on the division of residence, as defined by the US Census, and distance to the nearest hospital. We calculated the distance to the nearest hospital using the distance from the centroid of the postal code to the nearest facility on the basis of data from ESRI in 2010.^21^

We assigned temperature levels using the gridMET dataset, which provides daily estimated meteorological parameters on a 4 km-by-4 km scale.^22^ We aggregated these levels to postal codes and years. We created one variable for the warm season, which was the average temperature from April to the end of September, and one variable for the cold season, which was the average temperature from January to March and October to December of the same calendar year.

Observations with missing information were assumed to be missing at random and were excluded from analysis. These represented less than 1% of the data.

### Statistical analysis

Several papers^14,23,24^ have used propensity scores to analyse the association between air pollution and mortality. Briefly, the approach starts by first examining the association between exposure and confounders and uses that relationship to mimic a randomised trial, which makes the exposure independent from the confounders by creating a pseudopopulation in which individuals exposed to different concentrations of exposure are exchangeable with respect to the covariates.^25^ Specifically, we accounted for confounding via two mechanisms. First, with inverse-probability weights (IPWs) of exposure, and second, by adjusting for confounders in the outcome regression model. If either of the models is correctly specified, the estimated coefficient is unbiased.^24^ In the first step, we calculated stabilised IPWs for each exposure of interest using the following formula:

$$SW=\frac{\text{f}\left(\text{x}\right)}{\text{f}\left(\text{x|v}\right)}$$

In which x represents the exposure, v represents the covariates, and SW is the stabilised weight. The numerator was calculated as the probability density function of exposure from an intercept-only linear regression. The denominator was calculated as the probability density function of exposure given the covariates from a linear regression that included quadratic terms for all continuous confounders. To account for outliers, the highest percentile of weights was given the value at the 99th percentile, and the lowest percentile of weights was given the value at the 1st percentile.

In the second step, we ran a linear probability model to estimate the probability of death given the covariates and exposures of interest weighted by the IPWs calculated in the previous step.

$$Pr\;\left(\text{death}=1\right)=\beta_{0}+\beta x+s\left(v,\gamma \right)$$

In which x represents the exposure, v represents the vector of covariates, and γ represents the parameterisation of the covariates, which in this case included cubic terms for the other pollutants, temperature variables, age, and median household income. The equation was weighted by the weights calculated in the previous steps. For each exposure, we adjusted for the other two pollutants, both in the IPWs and in the outcome regression. A directed acyclic graph of the outcome regression is shown in the appendix (p 2). We used robust standard errors to account for the heteroscedasticity created by the inclusion of multiple observations from the same person and the use of a linear rather than logistic probability model. Because of our use of a linear probability model, the regression models estimate the absolute change in the risk of dying. To estimate the number of annual cases attributable to the exposures, we used the following formula:

Attributable cases = Risk difference per unit change per year×(Person−years of observation / duration of study)

Given that the dataset was large, we divided it randomly into groups and obtained the coefficients and standard errors using a fixed-effects meta-analysis of the group-specific results.

We also did single-pollutant analyses for comparison. We further identified subgroups vulnerable to potential environmental justice issues by looking at effect-measure modification (EMM) through stratification by Medicaid eligibility, race, sex, age group, quartiles of population density (as a measure of urbanicity), proportion of the population who identify as Hispanic, and median household income.

As a sensitivity analysis, we calculated E-values. Evidence-for-causality values identify the magnitude of the strength of the relationship that an unmeasured confounder would need to have with both the exposure and the outcome for its inclusion to change the effect estimate found to the null. A higher E-value is evidence of an analysis that is more robust to unmeasured confounding.^26^ Further, we ran the analyses adding cubic terms for all continuous predictors to the propensity-score model and quadratic terms in the outcome-regression model to see whether this adjustment would alter the effect estimates.

We evaluated the balance of covariates (after weighting) for the continuous variables by calculating the average absolute correlation coefficient between the exposures and the continuous variables, and tested whether using cubic terms would be better than quadratic terms.^27^

All data cleaning and statistical analyses were done in R statistical software version 3.5.1.

### Role of the funding source

The funders of this study had no role in study design, data collection, data analysis, data interpretation, or writing of the report.

## Results

The baseline characteristics of our population are presented in table 1. We identified approximately 40 million individuals with 267 million person-years of follow-up for PM_2·5_, 73 million individuals with 604 million person-years of follow-up for NO_2_, and 44 million individuals with 316 million person-years of follow-up for O_3_. Most Medicare participants were White and there were slightly more women than men.

The distribution of the exposure of interest in each dataset is shown in table 2 and the exposure distribution by demographic characteristics is shown in the appendix (pp 3–6). The correlation matrix for the exposures in each dataset is also presented in the appendix (p 7). The pollutants showed low-to-moderate correlation with each other, with correlation coefficient values of 0·11 to 0·59. Quartile definitions used in the EMM analyses are presented in the appendix (p 8).

The results of the primary analysis can be seen in table 3. Each single-unit increase in pollution concentration was associated with a 0·073% (95% CI 0·071–0·076) increase in the risk of death per year for PM_2·5_, 0·003% (0·003–0·004) for NO_2_, and 0·081% (0·080–0·083) for O_3_. This increase translated to approximately 11 500 attributable deaths for PM_2·5_, 1200 attributable deaths for NO_2_, and 15 100 attributable deaths for O_3_ per year for each unit increase in pollution concentrations. E values showed that the O_3_ and PM_2·5_ results were more robust to unmeasured confounding than the NO_2_ results. The single-pollutant results showed harmful effects for all pollutants (appendix p 8).

**Figure 1:**
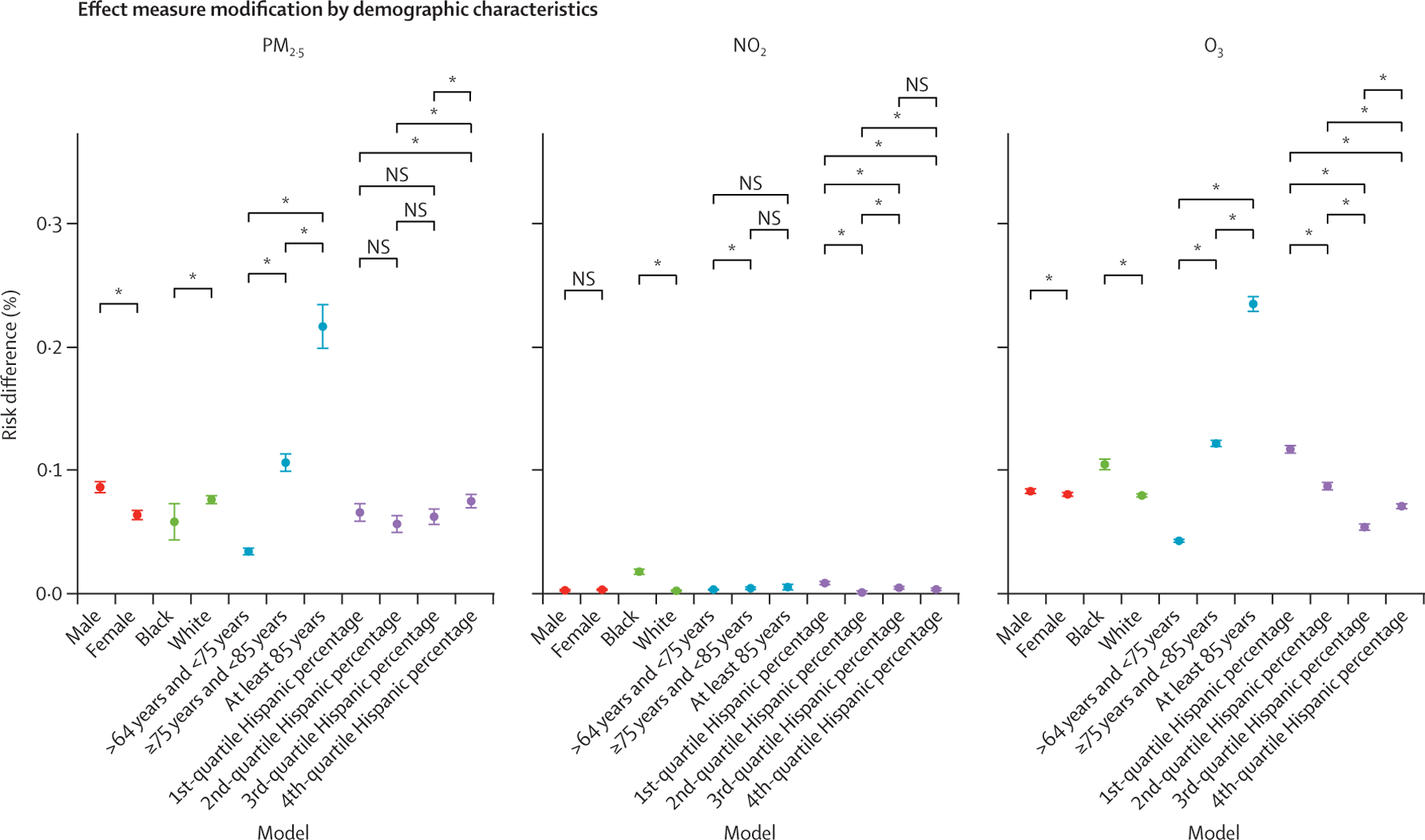
Effect-measure modification by demographic characteristics Risk-difference percentage (95% CI) change for each one-unit increase (1 μg per m^3^ for PM_2·5_ and 1 ppb for NO_2_ and O_3_) in annual pollutant concentrations among people who are always exposed to lower concentrations of air pollutants stratified by individual demographic characteristics. Pairwise comparisons of coefficients were done. Statistically significant differences (p<0·05) are indicated using asterisks. Ppb=parts per billion. PM_2·5_=particulate matter less than 2·5 μg/m³ in diameter. NO_2_=nitrogen dioxide. NS=non-significant. O_3_=ozone.

The results of the stratified analyses by demographic characteristics are presented in figure 1. Men (0·086%, 95% CI 0·082–0·091) were significantly more likely to die because of PM_2·5_ than women (0·064%, 0·060–0·068; p<0·0001). Individuals who identified as Black were more likely to die than those who identified as White because of NO_2_ (0·018%, 0·016–0·020 *vs* 0·002%, 0·002–0·003) and O_3_ (0·104%, 0·100–0·109 *vs* 0·079%, 0·078–0·081), whereas the reverse was true for PM_2·5_. People who were older were at greater risk of death than those who were younger, but this difference was more pronounced for PM_2·5_ and O_3_ than for NO_2_.

**Figure 2:**
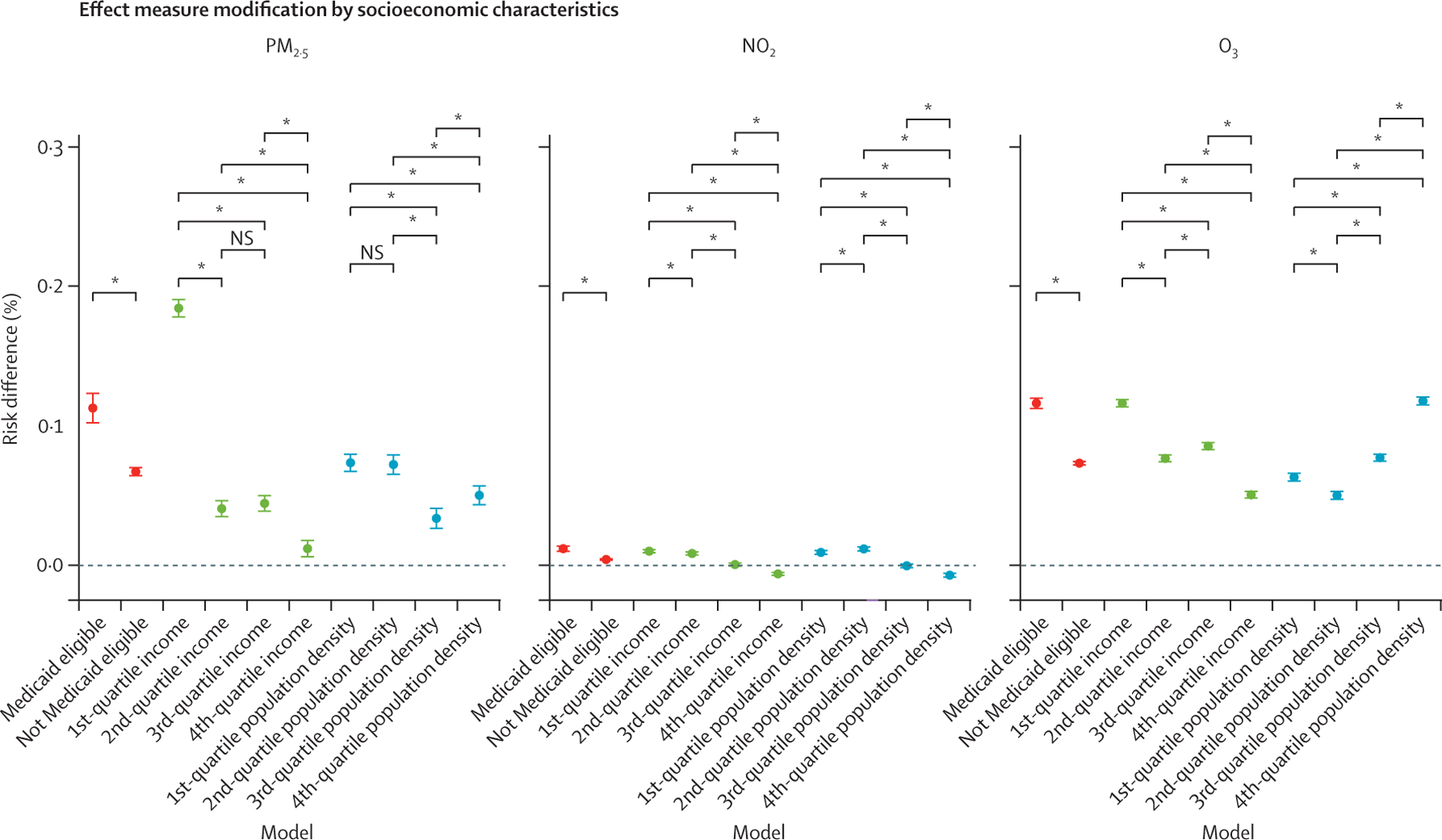
Effect Measure Modification by Socioeconomic Characteristics Risk-difference percentage (95% CI) change for each one-unit increase (1 μg/m³ for PM_2·5_ and 1 ppb for NO_2_ and O_3_) in annual pollutant concentrations among people who are always exposed to lower concentrations of air pollutants stratified by socioeconomic characteristics. Income refers to postal code-level median household income and population density refers to postal code-level population density. Pairwise comparisons of coefficients were done. Statistically significant differences (p<0·05) are indicated using asterisks. Ppb=parts per billion. PM_2·5_=particulate matter less than 2·5 μg/m³ in diameter. NO_2_=nitrogen dioxide. NS=non-significant. O_3_=ozone.

The results of the analyses stratified by socioeconomic status can be seen in figure 2. For all pollutants, people who were eligible for Medicaid were at a greater risk of death than those who were not eligible for Medicaid (0·113%, 0·103–0·124 *vs* 0·067%, 0·064–0·070 for PM_2·5_; 0·012%, 0·010–0·014 *vs* 0·004%, 0·004–0·005 for NO_2_; and 0·117%, 0·113–0·120 *vs* 0·073%, 0·072–0·075 for O_3_). This trend persisted when looking at income. People in the higher quartiles of postal code-level median household income had a lower risk of death than those in the lower quartiles of income.

**Figure 3:**
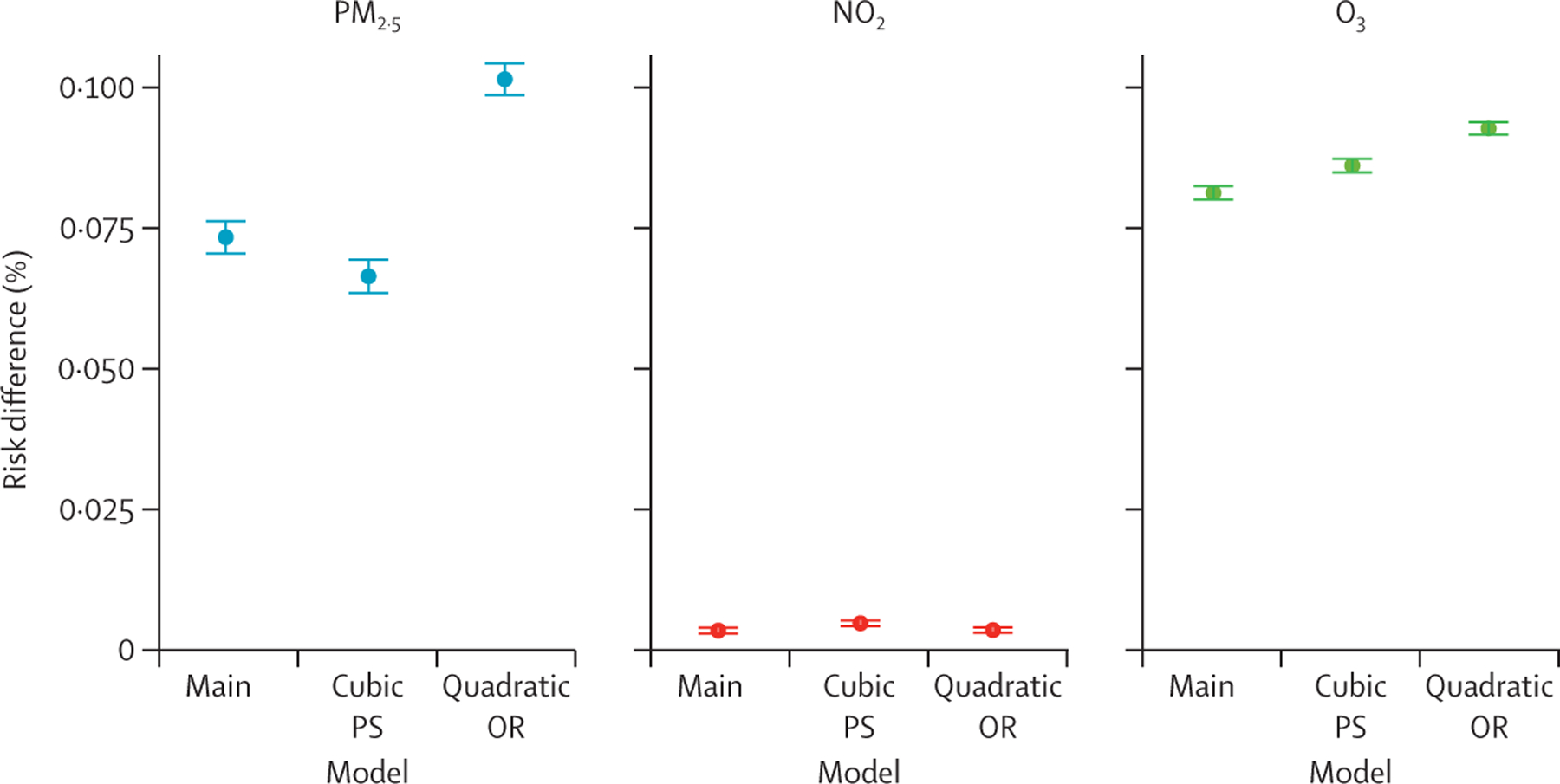
Model specification sensitivity analysis results Risk-difference percentage (95% CI) change for each one-unit increase (1 μg/m³ for PM_2·5_ and 1 ppb for NO_2_ and O_3_) in annual pollutant concentrations among people who are always exposed to lower concentrations of air pollutants. The figure shows a comparison of the main model used in our Article with a model that has cubic terms for the continuous variables in the PS model of the IPWs and a model that uses quadratic terms for age, median household income, temperature variables, and other pollutants in the OR. IPW=inverse probability weight. Ppb=parts per billion. PM_2·5_=particulate matter less than 2·5 μg/m³ in diameter. PS=propensity score. NO_2_=nitrogen dioxide. OR=outcome regression. O_3_=ozone.

The results of the sensitivity analyses can be seen in figure 3. Although changing the specification of the propensity-score model and the outcome regression altered the magnitude of the effect, the effect estimates were consistently positive. Our main model resulted in conservative estimates compared with several of the alternative specifications.

Finally, we checked the balance of covariates (appendix p 8). For all pollutants, weighting improved the balance of continuous covariates. We also ran the same analysis by adding cubic terms and it did not improve the balance of the continuous covariates for PM_2·5_ and NO_2_, although for O_3_, the improvement was minor.

## Discussion

Using a doubly-robust method, we found that among older individuals who were only exposed to lower concentrations of air pollutants during the study follow-up period, these pollutants increased the risk of death on an additive scale, after adjusting for other pollutants. This translated to tens of thousands of additional deaths per year per unit difference in exposure. We also confirmed that very old people and people with lower incomes were more vulnerable to air pollution. The results were made direclty interpretable by our use of an additive probability model instead of the more usual multiplicative model, which builds in interactions. This finding adds to the literature indicating that the effects of air pollution on mortality are causal, and that standards are inadequate.

The EPA released a decision regarding tightening the standards for ambient PM_2·5_. The EPA argued that “based on the available evidence, the Administrator has concluded that the current primary PM_2·5_ standards are requisite to protect public health, with an adequate margin of safety, from effects of PM_2·5_ in ambient air and should be retained, without revision.”^28^ Our results, which were based on current standards and were obtained using causal-modelling methodology, combined with the scientific assessment done by the EPA provide evidence that the US EPA Administrator’s decision for the annual PM_2·5_ standard was unjustified. Our findings suggest that reduction of air-pollution concentrations through stricter regulations would reduce mortality among older people, and given that the pollutants that we studied have several common sources, reductions in one pollutant would most likely reduce concentrations of the other pollutants as well. There are several areas in which intervention could occur that would affect all the pollutants of interest, including but not limited to stricter controls on industry and fossil-fuel electric-generating units, larger and more efficient catalysts on automobiles, city planning to promote active transport, and improved public transit. For example, the reduction of NO_2_ concentrations through higher-efficiency catalytic reduction or decreased vehicle use would likely reduce both PM_2·5_ and O_3_ as well.

Stratified analyses showed that people in higher age groups were at greater risk of death for all pollutants. Men were at greater risk of death caused by air pollution from PM_2·5_ and O_3_, and people who identified as Black had a higher risk of death caused by NO_2_ and O_3_. People with lower socioeconomic status tended to have a higher risk of death than those with higher socioeconomic status. The additional risk of mortality that we found in our stratified analyses for racial minorities and individuals with lower incomes raises concerns about environmental justice. These groups are historically disenfranchised and might suffer from exposure to air pollution and its adverse effects in a disproportionate manner.

Our results are not directly comparable to previous research, given that most studies have been done on a multiplicative scale or included higher exposures. A nationwide analysis of mortality in the Medicare cohort found that each increase of 10 μg per m³ in PM_2·5_ concentration was associated with a hazard ratio of 1·136 (95% CI 1·131–1·141) for observations with exposure concentrations of less than 12 μg per m³. The annual mortality in this cohort was 4·7 × 10^−2^, and a 1·28% increase in that mortality for an increment of 1 μg per m³ in PM_2·5_ would result in an additive increase of 6·0 × 10^−4^, or a 0·060% increase in the absolute risk of dying each year, similar to our result of 0·073%. That model, however, did not control for NO_2_.^4^ Another study looking at Medicare enrolees in the southeast region of the USA found that each one-unit increase in PM_2·5_ concentration was associated with a hazard ratio of 1·033 (95% CI 1·031–1·035) for concentrations of less than 12 μg per m³.^12^ A meta-regression looking at the dose-response relationship between long-term exposure to PM_2·5_ and mortality found a 2·4% (95% CI 0·8–4·0) increase in the risk of death for each increase of 1 μg per m^3^ in annual concentrations by restricting to studies with mean pollution concentrations of less than 10 μg per m³.^29^

Our study has several strengths. First, we used doubly-robust causal methodology to estimate our effects. Second, our outcome regression model was an additive model. This meant that our effect estimates could be used to directly estimate the number of deaths attributable to air pollution, without regard to the distribution of covariates. Third, our initial study population included everyone enrolled in Medicare. As such, we have sufficient power to detect effects in our analyses and our results have greater generalisability. Fourth, many of the previous papers using causal methods have only examined associations with PM_2.5,_^10,12,29^ whereas we examined PM_2·5_, NO_2_, and O_3_ simultaneously. Finally, we adjusted for several variables that could serve as proxies for socioeconomic status, which is the main confounder of concern in the air pollution–mortality relationship.

However, our study does have certain limitations. The causal implications of our study rely on the untestable assumption of no unmeasured confounding. We expect that should there be unmeasured confounding, it would most likely be caused by residual confounding through our inability to completely capture area-level socioeconomic status, although we did control for numerous variables in this category. We did try to assess the risk of potential unmeasured confounding by calculating E-values and found robust associations for PM_2·5_ and O_3_. Moreover, we cannot know whether the specification of either the propensity score or outcome regression is correct, although balance metrics showed that, at least for the continuous variables, there is minimal correlation with the exposure after weighting. Furthermore, we did not have data on comorbidities, which might have acted as mediators or effect modifiers of the air pollution and mortality relationship. Our pollutants were moderately correlated with one another, which might cause some collinearity, although given the large sample size, this problem is less likely to be of concern. Finally, the exposures were assigned on the basis of estimations from a prediction model, which will result in some measurement error. This measurement error is likely to be higher at extreme values for which there is limited monitoring data. We addressed this issue by restricting to lower concentrations and excluding the last three percentiles of observations. However, there is still potential for error. Further, NO_2_ and traffic-particle concentrations tend to decrease to urban background concentrations within 100–200 m of a busy road, and our NO_2_ and PM_2·5_ models do not capture the high exposure of people living within that distance of such a road. This is a limitation of our analysis.

Our study shows that for older individuals who are only exposed to low air-pollution concentrations during the follow-up period, an increase in the concentration of pollutants might still increase the risk of death. These results persisted in subgroup analyses, particularly for individuals with lower incomes. Regulators should consider setting annual standards for O_3_ and tightening regulations for PM_2·5_ and NO_2_.

## Supplementary Material

1

## Figures and Tables

**Table 1:** Demographic characteristics of Medicare enrollees

	PM_2·5_	NO_2_	O_3_
Number of individuals	40 422 099	72 769 408	44 430 747
Number of deaths	10 365 012 (25·6%)	28 594 819 (39·30%)	14 589 797 (32·8%)
Sex			
Female	21 692 819 (53·7%)	40 310 234 (55·39%)	24 283 979 (54·7%)
Male	18 729 280 (46·3%)	32 459 174 (44·61%)	20 146 768 (45·3%)
Race			
White	34 270 600 (84·8%)	61 205 628 (84·11%)	36 688 655 (82·6%)
Black	2 889 598 (7·1%)	6 525 454 (8·97%)	4 294 472 (9·7%)
Other	3 261 901 (8·1%)	5 038 326 (6·92%)	3 447 620 (7·8%)
Demographic characteristics by observations			
Number of person-years	267 111 005	604 486 421	315 767 917
Medicaid eligibility			
Yes	30 548 971 (11·4%)	76 308 600 (12·62%)	39 968 480 (12·7%)
No	236 562 034 (88·6%)	528 177 821 (87·38%)	275 799 437 (87·3%)
Age group			
Older than 64 years and younger than 75 years	178 420 757 (66·8%)	327 214 120 (54·13%)	189 968 539 (60·2%)
75 years or older and younger than 85 years	63 724 472 (23·9%)	198 374 334 (32·82%)	89 226 756 (28·3%)
At least 85 years of age	24 965 776 (9·3%)	78 897 967 (13·05%)	36 572 622 (11·6%)
Division			
Pacific	40 504 825 (15·2%)	81 087 764 (13·41%)	40 028 882 (12·7%)
Southwest central	29 691 495 (11·1%)	62 146 699 (10·28%)	31 144 181 (9·9%)
Northwest central	30 581 901 (11·4%)	43 995 791 (7·28%)	26 397 005 (8·4%)
Northeast central	21 649 239 (8·1%)	99 875 500 (16·52%)	59 337 855 (18·8%)
Southeast central	6 839 987 (2·6%)	35 755 594 (5·92%)	12 336 829 (3·9%)
Mountains	26 845 187 (10·1%)	34 138 704 (5·65%)	3 131 927 (1·0%)
South Atlantic	58 408 003 (21·9%)	124 552 351 (20·60%)	58 080 670 (18·4%)
Middle Atlantic	26 95 3775 (10·1%)	90 360 494 (14·95%)	55 836 410 (17·7%)
New England	25 636 593 (9·6%)	32 573 524 (5·39%)	29 474 158 (9·3%)

Data are n (%). PM_2·5_=particulate matter less than 2·5 μg/m³ in diameter. NO_2_=nitrogen dioxide. O_3_=ozone.

**Table 2:** Distribution of exposure of interest across person years

	Minimum	10th percentile	25th percentile	Mean	Median	75th percentile	90th percentile	Maximum
PM_2·5_, μg/m^3^	4·41	5·78	6·89	8·18	8·21	9·50	10·51	12·00
NO_2_, ppb	5·93	8·96	12·20	19·80	17·96	25·90	33·41	53·00
O_3_, ppb	31·93	36·14	39·41	41·94	42·53	44·72	46·73	50·00

Pbb=parts per billion. PM_2·5_=particulate matter less than 2·5 μg/m³ in diameter. NO_2_=nitrogen dioxide. O_3_=ozone.

**Table 3:** Main study results, including E values

	Risk difference (95% CI)*	Attributable increase in number of cases (95% CI)†	E value (multiplicative scale)
**Main analyses**			
PM_2·5_, μg/m^3^	0·073% (0·071–0·076)	11 540 (11 087–11 992)	1·062
NO_2_, ppb	0·003% (0·003–0·004)	1176 (998–1353)	1·013
O_3_, ppb	0·081% (0·080–0·083)	15 115 (14 896–15 333)	1·066
**Medicaid eligible**			
PM_2·5_, μg/m^3^	0·113% (0·103–0·124)	2032 (1843–2222)	1·079
NO_2_, ppb	0·012% (0·010–0·014)	540 (454–627)	1·024
O_3_, ppb	0·117% (0·113–0·120)	2741 (2653–2828)	1·080
**Not Medicaid eligible**			
PM_2·5_, μg/m^3^	0·067% (0·064–0·070)	9382 (8975–9789)	1·060
NO_2_, ppb	0·004% (0·004–0·005)	1332 (1175–1489)	1·014
O_3_, ppb	0·073% (0·072–0·075)	11 924 (11 724–12 123)	1·062
**Male individuals**			
PM_2·5_, μg/m^3^	0·086% (0·082–0·091)	6080 (5768–6393)	1·068
NO_2_, ppb	0·003% (0·002–0·004)	415 (296–534)	1·011
O_3_, ppb	0·083% (0·081–0·085)	6701 (6553–6850)	1·067
**Female individuals**			
PM_2·5_, μg/m^3^	0·064% (0·060–0·068)	5544 (5218–5871)	1·058
NO_2_, ppb	0·003% (0·003–0·004)	669 (538–800)	1·013
O_3_, ppb	0·080% (0·079–0·082)	8414 (8254–8574)	1·065
**Black individuals**			
PM_2·5_, μg/m^3^	0·058% (0·044–0·073)	525 (392–658)	1·055
NO_2_, ppb	0·018% (0·016–0·020)	542 (478–607)	1·030
O_3_, ppb	0·104% (0·100–0·109)	1766 (1693–1838)	1·075
**White individuals**			
PM_2·5_, μg/m^3^	0·076% (0·073–0·079)	10 505 (10 069–10 940)	1·064
NO_2_, ppb	0·002% (0·002–0·003)	747 (580–913)	1·011
O_3_, ppb	0·079% (0·078–0·081)	12 430 (12 221–12 640)	1·065
**Older than 64 years and younger than 75 years**			
PM_2·5_, μg/m^3^	0·034% (0·032–0·037)	3608 (3334–3881)	1·042
NO_2_, ppb	0·003% (0·003–0·004)	647 (555–738)	1·013
O_3_, ppb	0·043% (0·042–0·044)	4767 (4643–4891)	1·047
**75 years or older and younger than 85 years**			
PM_2·5_, μg/m^3^	0·106% (0·099–0·113)	3984 (3722–4247)	1·076
NO_2_, ppb	0·004% (0·003–0·005)	518 (406–630)	1·015
O_3_, ppb	0·121% (0·119–0·124)	6369 (6237–6501)	1·082
**85 years of age and older**			
PM_2·5_, μg/m^3^	0·216% (0·199–0·234)	3178 (2918–3438)	1·112
NO_2_, ppb	0·005% (0·003–0·008)	251 (142–360)	1·016
O_3_, ppb	0·234% (0·228–0·240)	5039 (4911–5168)	1·117
**1st-quartile median household income**			
PM_2·5_, μg/m^3^	0·185% (0·179–0·191)	7263 (7018–7508)	1·103
NO_2_, ppb	0·010% (0·009–0·011)	911 (814–1008)	1·022
O_3_, ppb	0·117% (0·114–0·119)	5413 (5293–5533)	1·080
**2nd-quartile median household income**			
PM_2·5_, μg/m^3^	0·041% (0·035–0·046)	1601 (1376–1825)	1·046
NO_2_, ppb	0·009% (0·007–0·010)	759 (662–857)	1·020
O_3_, ppb	0·077% (0·074–0·079)	3573 (3458–3688)	1·064
**3rd-quartile median household income**			
PM_2·5_, μg/m^3^	0·045% (0·039–0·050)	1751 (1534–1968)	1·048
NO_2_, ppb	0·001% (0–0·002)	NA‡	1·006
O_3_, ppb	0·086% (0·083–0·088)	3984 (3867–4102)	1·068
**4th-quartile median household income**			
PM_2·5_, μg/m^3^	0·012% (0·006–0·018)	474 (244–703)	1·024
NO_2_, ppb	−0·006% (−0·007 to −0·005)	NA‡	1·017
O_3_, ppb	0·051% (0·048–0·053)	2357 (2247–2468)	1·051
**1st-quartile population density**			
PM_2·5_, μg/m^3^	0·074% (0·068–0·080)	2898 (2656–3141)	1·063
NO_2_, ppb	0·009% (0·008–0·011)	827 (704–949)	1·021
O_3_, ppb	0·063% (0·061–0·066)	2946 (2816–3076)	1·058
**2nd-quartile population density**			
PM_2·5_, μg/m^3^	0·073% (0·066–0·080)	2849 (2575–3123)	1·062
NO_2_, ppb	0·012% (0·010–0·013)	1057 (933–1181)	1·024
O_3_, ppb	0·050% (0·048–0·053)	2338 (2208–2468)	1·051
**3rd-quartile population density**			
PM_2·5_, μg/m^3^	0·034% (0·027–0·041)	1328 (1048–1609)	1·042
NO_2_, ppb	0% (−0·002 to 0·001)	NA‡	1·004
O_3_, ppb	0·077% (0·075–0·080)	3597 (3482–3711)	1·064
**4th-quartile population density**			
PM_2·5_, μg/m^3^	0·050% (0·044–0·057)	1979 (1712–2246)	1·051
NO_2_, ppb	−0·007% (−0·008 to −0·006)	NA‡	1·019
O_3_, ppb	0·118% (0·115–0·121)	5491 (5362–5620)	1·081
**1st-quartile Hispanic percentage**			
PM_2·5_, μg/m^3^	0·066% (0·059–0·073)	2591 (2310–2872)	1·059
NO_2_, ppb	0·009% (0·007–0·010)	771 (652–889)	1·021
O_3_, ppb	0·117% (0·114–0·120)	5419 (5276–5561)	1·080
**2nd-quartile Hispanic percentage**			
PM_2·5_, μg/m^3^	0·057% (0·050–0·063)	2224 (1955–2492)	1·054
NO_2_, ppb	0·001% (0–0·002)	NA‡	1·007
O_3_, ppb	0·087% (0·084–0·090)	4033 (3895–4172)	1·068
**3rd-quartile Hispanic percentage**			
PM_2·5_, μg/m^3^	0·062% (0·056–0·069)	2454 (2209–2699)	1·057
NO_2_, ppb	0·005% (0·004–0·006)	432 (337–527)	1·015
O_3_, ppb	0·054% (0·051–0·056)	2495 (2379–2611)	1·053
**4th-quartile Hispanic percentage**			
PM_2·5_, μg/m^3^	0·075% (0·070–0·080)	2950 (2739–3160)	1·063
NO_2_, ppb	0·004% (0·003–0·005)	313 (222–405)	1·013
O_3_, ppb	0·071% (0·069–0·073)	3280 (3191–3368)	1·061

PM_2·5_=particulate matter less than 2·5 μg/m³ in diameter. NA=not applicable. NO_2_=nitrogen dioxide. O_3_=ozone.

* Per one-unit increase in pollutants per year (1 μg/m³ for PM_2·5_ and 1 ppb for NO_2_ and O_3_).

† Covariates included other pollutants, warm-season and cold-season temperatures, year, age, race, sex, Medicaid eligibility, region of residence, distance to nearest hospital, percentage of people who had an A1c exam, percentage of people who had an ambulatory-care visit, percentage of people who had a cholesterol exam, percentage of people who had an eye exam, percentage of women who had a mammogram, percentage of the population who are Black, percentage of the population who are Hispanic, population density per square mile, percentage of the population living below the poverty line, percentage of the housing occupied by the owners, median value of housing occupied by the owners, median household income, percentage of the population who did not graduate from high school, mean body-mass index, rate of smoking, and rate of lung cancer.

‡ Negative values on a probability scale are not logical. As such, attributable number of cases were not calculated.

## Data Availability

The data used in this study will not be made available publicly or to other researchers because of restrictions in the data-use agreement with the Centres for Medicare and Services (CMS). However, other investigators can apply to the CMS for their own data-use agreements to access the Medicare data.

## Research in context

### Evidence before this study

We searched PubMed for articles published in English with the following search terms: “air pollution”, “particulate matter”, “ozone”, or “nitrogen dioxide” and “mortality”. We limited the range of our search to the past 10 years and studies focusing on human adults. Several large cohort studies have found that the adverse health effects of long-term exposure to air pollution might be persistent even at lower concentrations, such as threshold concentrations set by the US Environmental Protection Agency and WHO. These cohorts are useful in that they include large subsets of individuals exposed to lower concentrations of air pollutants with enough power to detect effects. Studies that modelled dose-response curves did not find a pollution level at which no harm was observed.

### Added value of this study

In this study we used a doubly-robust, causal method to estimate the risk of mortality among people who were exposed to low concentrations of air pollutants over the study period.

We used a large dataset that gave us enough power to detect whether an association existed in demographic and socioeconomic subgroups. Furthermore, we assigned exposure on the basis of prediction models that were built on a fine scale with strong validation metrics.

All of the studied pollutants increased the risk of death in our study. We found that thousands of deaths could be attributed to small increases in annual air-pollution concentrations.

We also found an increased risk of death for people living in lower-income areas, suggesting that disparities exist in the adverse effects of air pollution.

### Implications of all the available evidence

This research adds greatly to the growing evidence that air pollution, even at low concentrations, might be harmful to human health and increase the risk of death. The state of evidence warrants a reconsideration of air-pollution standards.
