# Supplementary material for: Long-term effect of exposure to lower concentrations of air pollution on mortality among US Medicare participants and vulnerable subgroups: a doubly-robust approach
Source: Lancet Planet Health. Author manuscript; Available in PMC 2021 Oct 19. (PMC8525655; doi:10.1016/S2542-5196(21)00204-7)
Supplement: 1 [file NIHMS1746889-supplement-1.pdf]

THE LANCET
Planetary Health

Supplementary appendix

This appendix formed part of the original submission and has been peer reviewed. We post it as supplied by the authors.

Supplement to: Mahdieh Danesh Yazdi, Yan Wang, Qian Di, et al. Long-term effect of exposure to lower concentrations of air pollution on mortality among US Medicare participants and vulnerable subgroups: a doubly-robust approach. *Lancet Planet Health* 2021; **5:** e689–707.

Appendix

Figure S1. Directed Acyclic Graph of Proposed Exposure-Outcome Relationship

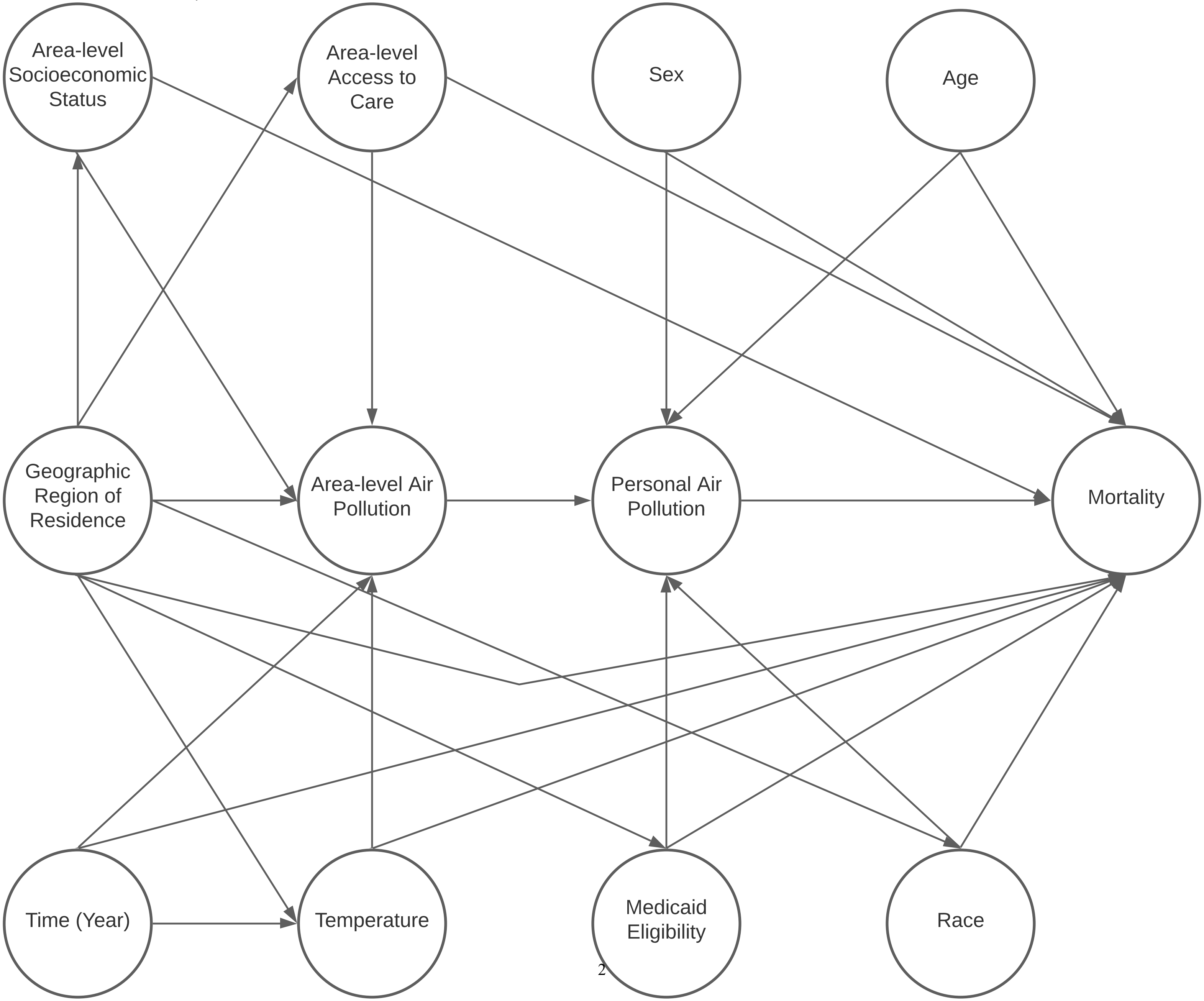

Table S1a. Exposure Distribution by Demographic Characteristics in the PM2.5 Dataset								
**Variable**	**Pollutant**	**Category**	**Min.**	**25th Percentile**	**Mean**	**Median**	**75th Percentile**	**Max.**
**Sex**	$PM_{2.5}$ (μg/m^3)	Female	4.41	6.91	8.19	8.22	9.51	12.00
		Male	4.41	6.87	8.17	8.20	9.48	12.00
	NO_2 (ppb)	Female	0.03	10.90	17.14	15.40	21.56	123.68
		Male	0.03	10.69	16.89	15.10	21.29	123.68
	O_3 (ppb)	Female	19.70	38.97	43.11	43.20	46.59	80.76
		Male	19.70	39.10	43.20	43.26	46.62	80.76
**Race**	$PM_{2.5}$	Black	4.41	7.67	8.68	8.69	9.75	12.00
		White	4.41	6.83	8.15	8.17	9.48	12.00
	NO_2 (ppb)	Black	0.03	12.07	18.94	17.92	24.44	123.68
		White	0.03	10.59	16.67	14.86	20.87	123.68
	O_3 (ppb)	Black	19.79	38.22	42.05	42.79	45.47	80.76
		White	19.70	39.30	43.30	43.28	46.68	80.76
**Age**	$PM_{2.5}$ (μg/m^3)	64<Age<75	4.41	7.08	8.29	8.35	9.56	12.00
		74<Age<85	4.41	6.59	7.97	7.91	9.34	12.00
		Age>84	4.41	6.55	7.95	7.87	9.32	12.00
	NO_2 (ppb)	64<Age<75	0.03	10.73	17.02	15.30	21.66	123.68
		74<Age<85	0.03	10.96	17.13	15.25	21.18	123.68
		Age>84	0.38	10.93	16.86	15.06	20.75	123.68
	O_3 (ppb)	64<Age<75	19.70	39.73	43.37	43.49	46.51	80.76
		74<Age<85	19.70	37.68	42.80	42.59	47.03	80.76
		Age>84	19.70	37.43	42.48	42.45	46.60	80.76

Table S1b. Exposure Distribution by Demographic Characteristics in the NO_2 Dataset

Variable	Pollutant	Category	Min.	25th Percentile	Mean	Median	75th Percentile	Max.
**Sex**	$PM_{2.5}$ (μg/m^3)	Female	0.51	8.08	10.14	9.93	12.12	30.92
		Male	0.51	7.93	9.97	9.77	11.91	30.92
	NO_2 (ppb)	Female	5.93	12.34	19.99	18.19	26.15	53.00
		Male	5.93	12.02	19.55	17.66	25.54	53.00
	O_3 (ppb)	Female	19.70	41.58	44.85	44.81	48.49	80.76
		Male	19.70	41.49	44.84	44.77	48.51	80.76
**Race**	$PM_{2.5}$	Black	0.51	8.95	11.02	10.73	12.87	30.92
		White	0.51	7.93	9.99	9.80	11.95	30.92
	NO_2 (ppb)	Black	5.93	14.12	23.07	22.44	30.93	53.00
		White	5.93	11.91	19.18	17.32	24.94	53.00
	O_3 (ppb)	Black	19.79	41.30	44.30	44.32	47.60	80.76
		White	19.70	41.70	44.98	44.91	48.60	80.76
**Age**	$PM_{2.5}$ (μg/m^3)	64<Age<75	0.51	7.94	9.99	9.78	11.93	30.92
		74<Age<85	0.51	8.13	10.21	10.01	12.21	30.92
		Age>84	0.51	8.04	10.06	9.84	11.96	30.92
	NO_2 (ppb)	64<Age<75	5.93	12.02	19.61	17.69	25.64	53.00
		74<Age<85	5.93	12.39	20.04	18.28	26.24	53.00
		Age>84	5.93	12.51	20.01	18.29	26.03	53.00
	O_3 (ppb)	64<Age<75	19.70	41.57	44.93	44.82	48.60	80.76

		74<Age<85	19.70	41.54	44.82	44.83	48.49	80.76
		Age>84	19.70	41.43	44.55	44.62	48.08	80.76

Table S1c. Exposure Distribution by Demographic Characteristics in the O_3 Dataset

**Variable**	**Pollutant**	**Category**	**Min.**	**25th Percentile**	**Mean**	**Median**	**75th Percentile**	**Max.**
**Sex**	$PM_{2.5}$ (μg/m^3)	Female	0.01	7.68	9.50	9.27	11.10	30.92
		Male	0.01	7.57	9.35	9.13	10.93	30.92
	NO_2 (ppb)	Female	0.01	11.30	19.60	17.10	26.11	127.63
		Male	0.01	10.90	18.88	16.32	25.04	127.63
	O_3 (ppb)	Female	31.93	39.43	41.95	42.53	44.71	50.00
		Male	31.93	39.38	41.94	42.54	44.73	50.00
**Race**	$PM_{2.5}$	Black	0.01	8.65	10.60	10.21	12.25	30.92
		White	0.01	7.50	9.28	9.10	10.87	30.92
	NO_2 (ppb)	Black	0.01	13.72	24.05	23.88	33.22	123.68
		White	0.01	10.77	18.33	15.85	24.03	127.63
	O_3 (ppb)	Black	31.93	39.65	42.10	42.57	44.67	50.00
		White	31.93	39.48	41.98	42.57	44.75	50.00
**Age**	$PM_{2.5}$ (μg/m^3)	64<Age<75	0.01	7.64	9.33	9.12	10.84	30.92
		74<Age<85	0.01	7.65	9.60	9.38	11.32	30.92
		Age>84	0.01	7.59	9.56	9.32	11.27	30.92
	NO_2 (ppb)	64<Age<75	0.01	10.77	18.55	16.09	24.44	127.63
		74<Age<85	0.01	11.65	20.31	17.79	27.50	127.63
		Age>84	0.01	11.87	20.56	18.08	27.80	127.63

	O_3 (ppb)	64<Age<75	31.93	39.72	42.17	42.78	44.94	50.00
		74<Age<85	31.93	38.92	41.57	42.14	44.35	50.00
		Age>84	31.93	39.04	41.64	42.23	44.37	50.00

**Table S2a. Correlation Matrix-$PM_{2.5}$ Data**			
	**$PM_{2.5}$**	**NO_2**	**O_3**
**$PM_{2.5}$**	1.00	0.23	0.11
**NO_2**	0.23	1.00	0.31
**O_3**	0.11	0.31	1.00

**Table S2b. Correlation Matrix-NO_2 Data**			
	**$PM_{2.5}$**	**NO_2**	**O_3**
**$PM_{2.5}$**	1.00	0.44	0.28
**NO_2**	0.44	1.00	0.17
**O_3**	0.28	0.17	1.00

**Table S2c. Correlation Matrix-O_3 Data**			
	**$PM_{2.5}$**	**NO_2**	**O_3**
**$PM_{2.5}$**	1.00	0.59	0.29
**NO_2**	0.59	1.00	0.12
**O_3**	0.29	0.12	1.00

Table S3. Quartile Definitions by Effect Modification							
**Variables**	**Pollutant**	**Min.**	**25th Percentile**	**Mean**	**Median**	**75th Percentile**	**Max.**
**Median Household Income**	$PM_{2.5}$ (µg/m^3)	0	39,565	49,381	54,617	64,294	334,368
	NO_2 (ppb)	0	38,186	48,256	53,403	63,336	338,701
	O_3 (ppb)	0	38,738	49,014	54,307	64,451	334,368
**Population Density**	$PM_{2.5}$ (µg/m^3)	0	130	751	2,338	2,873	311,353
	NO_2 (ppb)	0	184	999	3,153	3,316	361,621
	O_3 (ppb)	0	18	1,152	4,624	3,885	311,353
**% Hispanic**	$PM_{2.5}$ (µg/m^3)	0	0.025	0.063	0.13	0.162	1.00
	NO_2 (ppb)	0	0.021	0.052	0.124	0.144	1.00
	O_3 (ppb)	0	0.022	0.053	0.123	0.139	1.00

Table S4. Single Pollutant Results				
**Outcome**	**Pollutant**	**Risk Difference (%)**	**Lower 95% CI (%)**	**Upper 95% CI (%)**
**Main Analyses**	$PM_{2.5}$ (µg/m^3)	0.060	0.058	0.063
	NO_2 (ppb)	0.003	0.002	0.003
	O_3 (ppb)	0.090	0.089	0.091

Table S5. Absolute Average Correlation Coefficients for Continuous Covariates			
**Pollutant**	**Unweighted**	**Linear +Quadratic Terms**	**Linear+Quadratic+Cubic Terms**
**$PM_{2.5}$ (µg/m^3)**	0.087	0.047	0.049
**NO_2 (ppb)**	0.207	0.118	0.119
**O_3 (ppb)**	0.085	0.034	0.031
